# Effectiveness of MyKidEye in improving parents’ knowledge, attitude, and practice on children eye health care

**DOI:** 10.1371/journal.pone.0353767

**Published:** 2026-07-30

**Authors:** Nor Diyana Hani Ghani, Norliza Mohamad Fadzil, Zainora Mohammed, Mohd Harimi Abd Rahman, Rosilah Hassan, Hanif Farhan Mohd Rasdi

**Affiliations:** 1 Optometry and Vision Science Program, Centre for Rehabilitation and Special Needs Study (iCaRehab), Faculty of Health Science, University Kebangsaan, Malaysia; 2 Centre for Cyber Security, Faculty of Information Science and Technology, University Kebangsaan Malaysia, Bangi, Selangor, Malaysia; 3 Occupational Therapy Program, Centre for Rehabilitation and Special Needs Study (iCaRehab), Faculty of Health Sciences, Universiti Kebangsaan, Malaysia; University of Hafr Al-Batin, SAUDI ARABIA

## Abstract

**Background:**

Vision problem among children is the most common health concern worldwide. Parental involvement plays an important role in addressing these issues especially through their Knowledge, Attitude, and Practice (KAP) regarding vision care. MyKidEye is an innovative smartphone application that contain information on common vision problem in children, early signs and symptoms, and treatment options to enhance parent’s KAP. This study aims to assess the effectiveness of MyKidEye application in improving parental KAP related to children’s eye health care.

**Methods:**

This is a quasi-experimental study conducted from January to February 2023. The MyKidEye smartphone application was developed, and efficacy testing was performed. Parents from two schools who met the study criteria were selected and divided into two groups: the control group and the study group. Both groups completed the Parental Knowledge, Attitude, and Practice in Eye Problem among Children Questionnaire (PEPC-KAPQ) and their responses were recorded as pre-intervention data. Parents in the study group were given the MyKidEye application to be used for 4 weeks. Following a four weeks period, the PEPC-KAPQ was re-administered to both groups, and their responses were recorded as post-intervention data.

**Result:**

A total of 178 parents were enrolled in this study, with 89 parents in the control group and 89 in the study group. Statistical analysis was performed using two-way mixed ANOVA to evaluate changes in parental knowledge, attitude, and practice score between groups over time. The practice scores of parents in the study group showed significant improvement after using MyKidEye, compared to the control group (F (1,176) = 34.27, p < 0.01). However, no significant changes were observed in knowledge (F (1,176) = 0.02, p = 0.88) or attitude score (F (1,176) = 2.82, p = 0.10).

**Conclusion:**

MyKidEye application effectively improved parental practices related to children’s eye health care. It can serve as a valuable tool for improving and sustaining parents’ knowledge, attitudes, and practices regarding children’s eye health over a prolonged period, ultimately promoting better long-term eye care and awareness.

## Introduction

Vision plays a vital role in children’s development and overall quality of life, as it is responsible for nearly three-quarters of their learning process [1]. According to the World Health Organization (WHO), children account for approximately 29% of the 26 million individuals affected by vision problems worldwide [2]. When left untreated, vision problems can severely restrict children’s ability to engage fully in their education. Poor vision can disrupt their ability to participate in reading, writing, and other classroom activities, leading to challenges in grasping key concepts and skill. This not only affects their academic performance but can also hinder their social development, self-confidence, and long-term opportunities in life [3–5].

Parents are the primary caregivers responsible for ensuring their children receive timely eye care services [3,6–8]. However, some parents struggle to identify their child’s eye problems, as some conditions are asymptomatic, and children are often unaware of their poor vision [9,10]. Previous study reported that parents have poor knowledge and practices related to children’s eye health and their perceptions are often tainted with misconception [11]. While various programs and awareness campaigns have been implemented globally to educate parents about the importance of children’s eye health, including the use of guides, leaflets, games, and screenings, many parents still struggle to detect eye problems or seek early examinations and treatment for their children. Despite these efforts, the gap in awareness persists [12–15].

In recent years, the use of mobile applications has become increasingly widespread. The World Health Organization’s Global Observatory for eHealth defines mobile health (mHealth) as health and medical strategies supported by mobile technology, such as mobile phones. It involves the use of advanced functionalities and applications in addition to basic mobile services [16]. According to a literature review, smartphones are emerging as the primary platform for mHealth apps [17–19]. Research on the connection between smartphone use and children’s well-being has been steadily increasing [20]. The Malaysia Communication and Multimedia Commission [21] reported an increased in smartphone use for information seeking, rising from 89.4% in 2016 to 93.1% in 2018. This information-seeking behaviour also includes search related to health. The growing trend suggests that smartphones have become an increasingly important tool for accessing health-related information [22–26].

As a result, the MyKidEye application was developed as an innovative module for children eye health care, designed in the Bahasa Malaysia language. The app aims to equip parents with the knowledge, attitudes, and practices regarding common vision problems in children. MyKidEye is expected to raise awareness among parents about the importance of early eye examinations and treatment to prevent more severe issues, ultimately reducing the risk of avoidable blindness in children. Over time, the MyKidEye application is anticipated to enhance parents’ understanding and encourage proactive eye health behaviour, ensuring children receive timely care at the first signs of vision problem. Consequently, this study aimed to evaluate the effectiveness of MyKidEye in improving parents’ knowledge, attitudes, and practices on children eye health care.

## Materials and methods

This study was approved by the Research Ethics Committee of Universiti Kebangsaan Malaysia (JEP-2020–739) and Ministry of Education Malaysia (KPM.600–3/23-eras (11562). This research was conducted in accordance with the ethical standard outlined in the Declaration of Helsinki.

### Study design

This was a quasi-experimental study conducted in two schools in Kuala Lumpur. Kuala Lumpur was selected because the research was done during the Movement Control Order (MCO), which restricted interstate travel. Wangsa Maju district was randomly selected from the 11 districts in Kuala Lumpur. Two public primary schools (age between 7–12 years old) were chosen using convenience sampling, with a maximum distance of 15 kilometres between them. This approach followed Grossman’s theory, which suggests that income, lifestyle, education, and location affect healthcare access [27]. This design was aimed to minimizing socio-economic factors that could influence the study result.

### Subject

The subjects were selected through random sampling to avoid systematic bias. The inclusion criteria for selecting the participants were as follows: parents or guardian must be able to understand the Bahasa Malaysia language; have a child aged between 7 and 12 years old; and they must own a smartphone. These criteria ensured that the participants could understand the study materials and able to engage with the MyKidEye application effectively.

### Procedures

Parents who met the eligibility criteria were selected to participate in this study. A face-to-face session was conducted with parents. Informed consent was obtained by explaining the purpose, process, risk, and benefits of the study in simple language that participants could understand. Parents were informed that participation was voluntary, that they could withdraw at any time without penalty, and that their information would be kept confidential. The participants were aware that their clinical information were reported in a medical publication collectively. Time was given for questions, and only parents who agreed and signed the consent form were included in the study.

Parents from one school were assigned to the control group, while parents from another school were assigned to the study group. The Parental Knowledge, Attitude, and Practice in Eye Problem among Children Questionnaire (PEPC-KAPQ) [28] was distributed to the parents who agreed to participate. Completed responses were collected and recorded in Microsoft Office Excel, along with demographic information such as age, gender, and ethnicity. This information was recorded as pre-intervention data.

**Fig 1:**
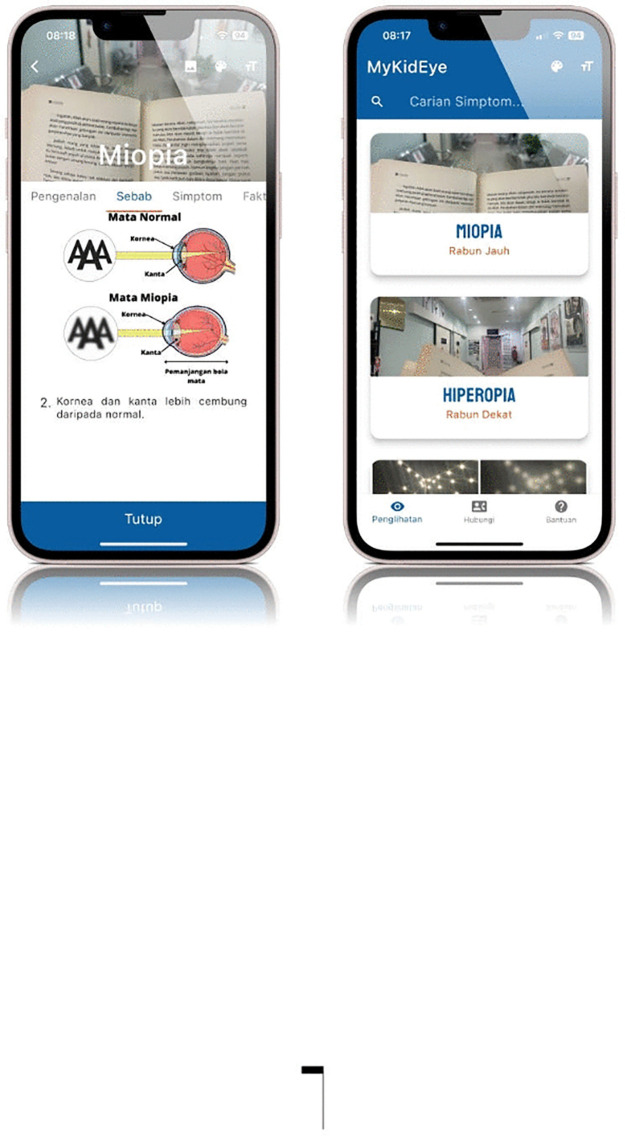
Information in MyKidEye smartphone application.

All parents in the study group were provided with a link to the MyKidEye application via WhatsApp. Before using the app, a face-to-face session was conducted with parents to give a brief explanation of the purpose, features, and instruction on how to navigate and read the information about children’s eye health (Fig 1). They were asked to use it for four weeks. They received two reminders during this period to encourage continued use of the app, exploring all features and reading the content thoroughly. Previous studies have shown that a four-week period is sufficient for users to adapt to and interact with a new app [29]. This approach aligns with previous research reporting that acceptance of a new app depends on actual use, including frequency and duration of interaction. By allowing adequate time, users can explore and fully utilize the app’s features before the assessment [29]. Parents in the control group did not receive any intervention. After four weeks, the PEPC-KAPQ was redistributed to the parents in both groups. The parents’ responses were recorded as post-intervention data.

### Tools

The PEPC-KAPQ consists of three domains: knowledge, attitude, and practice, with a total of 52 items (knowledge 26 items; attitude: 17 items; practice: 9 items). The knowledge domain was measured using a “Yes” or “No” scale, where a “Yes” response was scored as 1 and a “No” response was scored as 0. The attitude domain was measured using a 4-point Likert scale: “Strongly agree” = 3, “Agree” = 2, “Disagree” = 1, and “Strongly disagree” = 0. The practice domain was measured using the following scores: “Very often” = 3, “Often” = 2, “Rarely” = 1, and “Never” = 0. The PEPC-KAPQ was developed in Bahasa Malaysia and has good validity and reliability [28]. The scoring for each domain: knowledge (min: 0, max: 26); attitude (min: 0, max 68) and practice (min: 0, max: 36).

Examples of questionnaire items included: for the knowledge domain, “Are the following signs associated with vision problems among children? (e.g., red eyes)” with response options of *Yes* or *No*. For the attitude domain, participants were asked to rate their agreement with the statement, “Eye examinations are important for your child,” using a four-point Likert scale (*strongly agree, agree, disagree, strongly disagree*). For the practice domain, participants were asked, “How often do you monitor your child’s gadget usage?” with response options of *very often, often, rarely,* and *never*

### Statistical methods

Statistical analysis was performed using IBM SPSS Statistics version 21. Descriptive statistics were used to summarise the participants’ demographic characteristics and the score of the knowledge, attitude, and practice domains. The normality of the data distribution was assessed using the Shapiro-Wilk test. As the data were normally distributed (*p* < 0.05), parametric statistical tests were applied. An independent t-test was conducted to compare the pre-intervention PEPC-KAPQ score between the control and study groups. Effect sizes for significant differences were calculated using Cohen’s d [30]. Additionally, a two-way mixed ANOVA was performed to examine difference between the control and study groups, focusing on the main effects and interactions between group (control vs study) and time (pre vs post intervention) for knowledge, attitude, and practice scores following the MyKidEye intervention.

## Results

### Subject demographics

A total of 178 subjects, aged between 29 and 57 years (with a mean age of 41.79 ± 5.50 years), participated in this study. There were 85 male participants (47.8%) and 93 female participants (52.2%). Among them, 169 subjects (94.9%) were of Malay ethnicity, 3 (1.7%) were of Indian ethnicity, and 6 (3.4%) were from other ethnic backgrounds.

The control and study groups each consisted of 89 subjects, with the control group from school A and the study group from school B. The distance between the two schools was 9.9 km, which is within the required range to ensure parents from both schools belong to similar socioeconomic categories [27].

### Knowledge, Attitude, and Practice (KAP) scores preintervention

The results showed a significant difference in the knowledge score between two group (Table 1). However, the effect size (Cohen’s d) for the knowledge score was small, at 0.38, suggesting the difference is moderate and likely to have a minimal impact on the intervention (Cohen, 1988).

**Table 1:** Independent T-test (n = 178).

Domain	Mean ± SD		*P* value	Effect size (Cohen’s d)
	Control	Study		
Knowledge	38.92 ± 7.15	41.75 ± 7.70	0.01	−0.38
Attitude	55.53 ± 7.12	57.12 ± 6.79	0.13	−0.23
Practice	55.53 ± 7.12	15.12 ± 4.25	0.23	0.16

### The effectiveness of MyKidEye

In this study, a two-way mixed ANOVA was conducted to examine the difference between the control group and the study group, focusing on the main effects and interactions between groups (control and study) and time (pre- and post-intervention) concerning knowledge, attitude, and practice scores following the MyKidEye intervention.

### Knowledge score

The results showed a significant main effect for the group, [F (1,176) = 10.06, p < 0.01, ηp2 = 0.05], with the study group scoring higher (M = 41.75, SD = 7.70) compared to the control group (M = 38.92, SD = 7.15). However, the analysis revealed that the main effect for time was not significant, [F (1,176) = 0.02, p = 0.88, ηp2 < 0.01], indicating no significant change in knowledge scores from pre- to post-intervention for either group.

Additionally, the interaction effect between group and time was also not significant, [F (1,176) = 0.04, p = 0.84, ηp2 < 0.01]. These findings suggest that there was no significant difference in the changes of knowledge scores between the study and control groups over the study period. Table 2 presents the results of the two-way mixed ANOVA for pre- and post-intervention knowledge scores.

**Table 2:** Knowledge: Two-way mixed ANOVA for pre- and post- intervention (n = 178).

Group		Knowledge Score		Main effect within group			Main effect between group		Interaction Time*Group	
	Pre *(M ± SD)*		Post *(M ± SD)*		*P*	*ηp* ^*2*^	*p*	*ηp* ^*2*^	*p*	*ηp* ^*2*^
Control	38.92 ± 7.15		38.89 ± 6.99		<0.01	0.05	0.88	<0.01	0.84	<0.01
Study	41.75 ± 7.70		41.99 ± 8.19							
Total	40.34 ± 7.54		40.44 ± 8.14							

### Attitude score

The results indicated that comparison of attitude scores within and between the groups showed no significant main effect for the group, [F (1,176) = 2.90, p = 0.09, ηp² = 0.02], nor for time, [F (1,176) = 2.82, p = 0.10, ηp² = 0.02]. This suggests that changes in attitude scores from pre- to post-intervention were not significant in either group.

The interaction effect between group and time was not significant, [F (1,176) = 0.06, p = 0.80, ηp² < 0.01]. These findings show that the comparison of attitude score changes between the study and control groups throughout the study period was not significant. The results of the two-way mixed ANOVA for pre- and post-intervention attitude scores are presented in Table 3.

**Table 3:** Attitude: Two-way mixed ANOVA for pre- and post- intervention (n = 178).

Group		Attitude Score		Main effect within group			Main effect between group		Interaction Time*Group	
	Pre *(M ± SD)*		Post *(M ± SD)*		*P*	*ηp* ^*2*^	*p*	*ηp* ^*2*^	*p*	*ηp* ^*2*^
Control	55.53 ± 7.12		56.63 ± 7.18		0.09	0.02	0.10	0.02	0.80	<0.01
Study	57.12 ± 6.79		57.94 ± 6.47							
Total	56.33 ± 6.98		56.33 ± 6.85							

### Practice score

The results of practice score also showed that the main effect for the group was not significant, [F (1,176) = 0.02, p = 0.09, ηp2 < 0.01]. However, the main effect for time showed a significant difference, [F (1,176) = 34.27, p < 0.01, ηp2 = 0.16]. For the study group, post-intervention practice scores (M = 16.48, SD = 3.38) were higher compared to pre-intervention scores (M = 15.12, SD = 4.25), while in the control group, both pre-intervention (M = 15.78, SD = 3.73) and post-intervention (M = 15.99, SD = 3.66) practice scores were almost the same.

The interaction effect between group and time was also significant, [F (1,176) = 18.19, p < 0.01, partial η2 = 0.09]. These results indicate a significant change in practice scores between the study and control groups over the study period. Table 4 shows the results of the two-way mixed ANOVA for pre- and post-intervention practice scores.

**Table 4:** Practice: Two-way mixed ANOVA for pre- and post- intervention (n = 178).

Group		Practice Score		Main effect within group			Main effect between group		Interaction Time*Group	
	Pre *(M ± SD)*		Post *(M ± SD)*		*P*	*ηp* ^*2*^	*P*	*ηp* ^*2*^	*p*	*ηp* ^*2*^
Control	15.78 ± 3.73		15.99 ± 3.66		0.09	0.01	<0.01	0.16	<0.01	0.09
Study	15.12 ± 4.25		16.48 ± 3.38							
Total	15.45 ± 4.00		16.24 ± 3.52							

## Discussion

### Knowledge score

In this study, the results revealed a statistically significant difference in pre-intervention knowledge scores between the control and study groups. However, the effect size (Cohen’s d) for knowledge scores was small (0.38). This indicate a moderate difference that may not reflect a substantial impact of the intervention [30]. Thus, it was assumed that both groups had comparable level of knowledge prior to the intervention. Despite this, the increase in post-intervention knowledge scores within both groups was not statistically significant, and between groups comparison also demonstrated no significant difference. These findings suggest that the use of MyKidEye did not lead to a significant improvement in parental knowledge within the study group.

Several factors may have influenced the knowledge scores, including the usage of the MyKidEye by parents. Previous studies indicated that parents who use the application more frequently tend to demonstrate greater improvement in knowledge and associated behavioral changes [29,31–33]. Other studies have reported that consistent and active use of mobile application is associated with improved knowledge through mastery of the information. In contrast, infrequent or inconsistent usage may prevent users ability to fully understand and utilize the content of the application, thereby limiting significant improvements in knowledge [29,31–33]. In this study, the duration of parental usage of the MyKidEye application was not recorded and was based solely on self-reported data provided by the participants.

Another factor that may influence the effectiveness of MyKidEye is the way parents engage with the app. The way smartphone applications are used can significantly impact how information is received and comprehended [34,35]. The four-week periods given may not be sufficient for parents with limited technological proficiency. They may encounter difficulties in navigating and fully utilizing the features offered by MyKidEye, thus require more time. In addition, MyKidEye lacks a mechanism for assessing whether users are engaging with the app to its full potential.

Differences in parents’ interest or willingness to learn may also influence the effectiveness of the application. Some parents may have a strong motivation to acquire knowledge regarding vision problems in children and actively use MyKidEye, while others may be less motivated or interested. Variations in interest and engagement among parents can lead to differing outcomes in term of how effectively the app enhances parental knowledge [31]. This is may be because knowledge acquisition require comprehensive, reflective approach and active interaction with the learning material [31,33,36].

The rate at which information is received and processed can also influence the effectiveness of MyKidEye application. The ability of each individual to absorb and comprehend information varies, each person learning at a different pace. Some parents may be able to grasp and apply the information quickly, while others may require more time to fully comprehend and understand the content [32].

### Attitude score

The findings showed that the change in pre- and post-intervention attitude scores for both the control and study groups were not significant. Similarly, these findings indicate that the improvement in attitude scores in the study group was not significant and did not differ from the control group.

Changing attitudes involves a complex process that requires time for reflection and deeper cognitive adjustment. A period of 4 weeks may have been too short for measuring attitude change. Attitudes involve not only changes in knowledge but also require a transformation in thinking and a more comprehensive perspective. Therefore, long-term effects may not be easily detected within such a short timeframe, as attitude changes typically require more time to develop and consistently influence behavior [37,38].

Changing in attitude are also influenced by existing knowledge and the extent to which individuals are able to apply that knowledge into practice [36]. Parent with a greater and deeper understanding of health-related issues are more likely to adopt behaviour that reflect their knowledge, which ultimately lead to a change in attitude towards actively monitoring their child’s development. Although the participants shared a similar socioeconomic background, variations in personal experiences and individual beliefs may have contributed to differences in their attitudes and perceptions [39].

Social influences also play a role in shaping parents’ attitudes. Support or encouragement from family, friends, or the community can significantly impact how parents perceived and use the app. Parents who receive positive reinforcement from those around them may be more motivated to engage with the app and use it more effectively.

The pre-intervention attitude score was already relatively high at the beginning of the study, which may explain the absence of a statistically significant change following the intervention. When baseline scores are high, the potential for further improvement becomes limited, a phenomenon commonly referred to as the ceiling effect. From a theoretical perspective, behaviour change models suggest that individuals who already possess positive attitudes may not demonstrate substantial attitudinal shifts after an intervention because their attitudes have already reached a favourable level. Instead, the intervention may primarily facilitate the translation of existing knowledge and attitudes into actual behaviour, which could explain the significant improvement observed in the practice domain [40].

### Practice score

The results of this study found a significant increase in practice scores post-intervention for the study group, whereas the increase in practice scores for the control group was not statistically significant. The comparison of changes in practice scores between the two groups was also significant following the use of MyKidEye, indicating potential effectiveness in improving parental practices related to children’s eye health.

These findings are consistent with previous studies indicating that mHealth apps tend to be more effective in behaviour change compared to improving knowledge and individual attitudes [33,37,41]. This may be because mHealth apps often incorporate practical interactions features and provide continuous motivation for behavioral change. Furthermore, studies by Hashemian et al. (2015) and Zhao et al. (2016) also support the view that mHealth apps help individuals adopting healthier lifestyles. These applications are believed to encourage users to take actions that can lead to more positive and sustained behavioral changes. In the present study, the observed improvement in practice scores could be attributed to the content provided within the MyKidEye, which includes information on treatment, effects, and complications for common vision problems in children. Although there was no significant improvement in knowledge scores, parents in the study group were exposed to treatment related information of children with vision problems. It is likely that the exposure to this information influence parents’ response to the practice section of the survey, resulting in a significant increase in the practice scores compared to the control group.

MyKidEye application also incorporate information regarding symptoms of common vision problems in children. For example, the section on myopia within the application highlighting symptoms such as sitting too close to screens and squinting. This information may have enabled parents in the study group to identify vision issues in their children and take appropriate action, in contrast to those in the control group. Additionally, the apps use simple language, which may have facilitated parents’ understanding, enabled them to make the right decisions and act quickly, thus positively affecting their practices. These findings are consistent with previous research, suggesting that when individuals received clear and practical information, they are more likely to respond quickly and effectively, adjusting their behaviour accordingly [31,33].

The willingness to change in term of practices, knowledge, and attitudes can vary significantly. Changes in practices can occur more rapidly when clear, actionable steps are provided [29,32,37,38]. MyKidEye provides information such as definitions of common vision problems in children, their symptoms, treatments, and potential effects, making it easier for parents to access relevant information. MyKidEye also allows parents to directly contact optometrists and schedule appointments, enabling them to make informed decisions and take immediate action for prevention or treatment, which ultimately accelerates changes in their practices.

Furthermore, the distinction between short-term and long-term effects plays an important role in the process of improving knowledge, changing attitudes, and practices. Changes in practices are typically achievable in a shorter time frame when practical information is provided, as these actions can be implemented immediately. When parents are given specific and clear guidance on eye care, they are more likely to apply it immediately, leading to observable changes in their practices. In contrast, improvement in knowledge and changes in attitudes require more time, reflection and effort [29,32,37,38]. As a result, MyKidEye appears to have greater influence on parental practices than on knowledge and attitudes, as the latter involve more detailed and complex approach of learning and behaviour change.

## Limitation

This study has several limitations that should be considered when interpreting the findings. First, the duration and frequency of parental usage of the MyKidEye app were not objectively recorded, and solely relied on parents’ self-reports. Second, the study was conducted in only two schools within one district of Kuala Lumpur, which may limit the generalizability of the results. Third, the MyKidEye app did not include the function to track detailed engagement metrics, such as time spent on specific content. Therefore, the actual usage time of the apps by parents was not recorded. Finally, parents in the study group received two reminders via WhatsApp, which may have influenced their usage time of the apps and this could differ in real-world implementation. Future research could address these limitations by incorporating objective usage tracking, expanding to more diverse populations, and exploring strategies to maintain engagement.

## Conclusion

In conclusion, MyKidEye application was effective in improving parental practices related to seeking eye examination and treatment for their children. The finding demonstrate that digital tools can support parents in taking timely action to address their children’s vision needs. By encouraging proactive eye care practice, the app has the potential to contribute to the prevention of avoidable childhood vision problems and support early detection of vision issues. There results highlight the value of app-based interventions in promoting positive health behaviours among parents and underscore the importance of integrating such tools into broader child eye health initiatives.

### Implication for future study

The updated version of the MyKidEye application could include a Q&A feature that allows parents or guardians to interact directly with experts. This feature would enable users to ask questions or raise concern to eye health professionals, thus improving interactivity and support for parents and guardians.

## Supporting information

S1 Data Data of participants on questionnaire responses. (XLSX)
